# Identification of a New Intronic BMPR2-Mutation and Early Diagnosis of Heritable Pulmonary Arterial Hypertension in a Large Family with Mean Clinical Follow-Up of 12 Years

**DOI:** 10.1371/journal.pone.0091374

**Published:** 2014-03-12

**Authors:** Katrin Hinderhofer, Christine Fischer, Nicole Pfarr, Justyna Szamalek-Hoegel, Mona Lichtblau, Christian Nagel, Benjamin Egenlauf, Nicola Ehlken, Ekkehard Grünig

**Affiliations:** 1 Institute of Human Genetics, University of Heidelberg, Heidelberg, Germany; 2 Centre for pulmonary hypertension of the Thoraxclinic, University Hospital of Heidelberg, Heidelberg, Germany; Vanderbilt University Medical Center, United States of America

## Abstract

**Background:**

Mutations in the bone morphogenetic protein receptor 2 (*BMPR2*) gene can lead to hereditary pulmonary arterial hypertension (HPAH) and are detected in more than 80% of cases with familial aggregation of the disease. Factors determining disease penetrance are largely unknown.

**Methods:**

A mean clinical follow-up of 12 years was accomplished in 46 family members including echocardiography, stress-Dopplerechocardiography and genetic analysis of TGF-β pathway genes. Right heart catheterization and RNA-analysis was performed in members with pathological findings.

**Results:**

Manifest HPAH was diagnosed in 8 members, 4 were already deceased, two died during the follow-up, two are still alive. Normal pulmonary artery systolic pressure at rest but hypertensive response to exercise has been identified in 19 family members. Analysis of *BMPR2* transcripts revealed aberrant splicing due to an insertion of an intronic Alu element adjacent to exon 6. All HPAH patients and 12 further asymptomatic family members carried this insertion. During follow-up two family members carrying hypertensive response and the Alu insertion developed manifest HPAH.

**Conclusion:**

This is the first report of an intronic BMPR2 mutation due to an Alu element insertion causing HPAH in a large family which has been confirmed on RNA-level. Only those members that carried both hypertensive response and the mutation developed manifest HPAH during follow-up. Our findings highlight the importance of including further methods such as RNA analysis into the molecular genetic diagnostic of PAH patients. They suggest that at least in some families hypertensive response may be an additional risk factor for disease manifestation and penetrance.

## Introduction

Pulmonary arterial hypertension (PAH) can be idiopathic (IPAH), heritable (HPAH) or associated with other conditions (APAH) [1] and is usually not detected before patients are severely affected with symptoms according to WHO-functional class III–IV and reduced prognosis [2]. Therefore, it might be of great value to identify the disease at an early stage.

Since 2000 in HPAH-patients and families several mutations of genes of the transforming growth factor beta (TGF-β) superfamily of receptors have been found as in the bone morphogenetic protein receptor 2 (*BMPR2*) gene [3]–[5], *Activin A receptor type II-like 1* (*ACVRL1*, also called *ALK1*) [6], *Endoglin* [7], and *SMAD9* [8] (also called SMAD8).

However, the major genetic determinant underlying HPAH are germline heterozygous mutations of the *BMPR2* gene on chromosome 2q33 that account for approximately 80% of patients with a known family history of PAH and 20% of apparently sporadic cases [9], [10]. More than 300 independent *BMPR2* mutations have been detected so far which are widely distributed across the 13 exons of the gene [5]. Today, standard genetic screening methods focus on the sequencing of the exonic coding regions of the *BMPR2*, *Alk1, Endoglin*, and *SMAD9* genes [11]. Intronic regions have not been studied systematically so far, though they might harbour disease causing mutations.

Although HPAH is a monogenetic disease with autosomal dominant inheritance [12] only ∼20% of BMPR2-mutation carriers will develop the disease due to an incomplete age and gender related penetrance [12], [13]. The underlying factors of the incomplete penetrance are yet unknown. Thus, in HPAH-families there are asymptomatic gene carriers with an approximately 20%-risk to develop manifest HPAH during their life span who might potentially be detected at an early stage of disease. Regular echocardiography and genetic counseling have been recommended in several PH-guidelines [1], [14], [15]. However, reports on follow-up assessments in these families analyzing the natural course of the disease and clinical signs of an early manifestation are lacking. So far there are no systematic prospective long-term follow-up studies which evaluated the usefulness of screening-assessments in these families. In one case, onset of HPAH was detected by follow-up cardiopulmonary exercise testing in an initially healthy relative with BMPR-2 mutation [16].

We previously described a large German family (S965) with 7 affected members in 3 generations [17]. This family had no identifiable mutations in *BMPR2* gene by exonic sequencing or multiple ligation-dependent probe amplification, although linkage to chromosome 2q32-33 has been detected [18]. At baseline, several members of this family with no signs of manifest PAH at rest showed hypertensive pulmonary artery systolic pressure (PASP) response to exercise (HR), measured by stress-Dopplerechocardiography [17], [18]. In a large multicenter study similar findings have been obtained in other PAH-families. In this study HR during exercise and hypoxia was identified more frequently in relatives of PAH patients than in healthy controls and was associated with a significantly higher proportion of BMPR2-mutations [19]. It was hypothesized, that this phenotype might constitute a risk factor for the development of manifest PAH in some asymptomatic relatives.

The aim of this study was to extend the molecular genetic analyses in this family to the candidate genes *ALK1*, *ENG*, or *SMAD9* and to RNA analysis of the *BMPR2* gene and to explore if manifest PAH can be clinically detected at an early stage in further family members by regular non-invasive follow-up assessments. Furthermore, the natural clinical course of asymptomatic mutation carriers and HR-members should be analysed.

## Materials and Methods

### Study population and design

**Figure 1:**
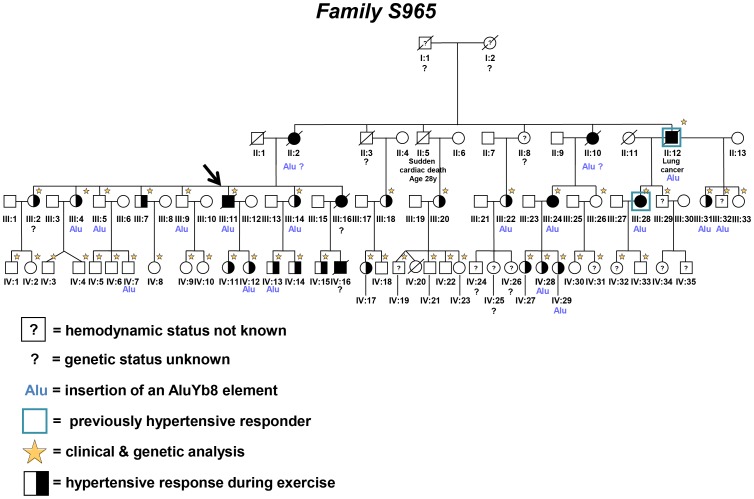
Pedigree of the large German family. This figure represents the pedigree tree of the German family analysed in this study. The index patient of the family is marked by an arrow. All family members with manifest PAH are shown in black. Healthy family members have open symbols and those who were heterozygous for the identified mutation are marked with “Alu”. Those family members with hypertensive response due to exercise have half-filled symbols. Family members with unknown hemodynamic status have open symbols with a question mark inside. A question mark below the pedigree ID indicates that the genetic status in this family member is unknown. An open blue square marks the family members which presented at the beginning of the follow-up with hypertensive response due to exercise and changed their status to manifest PAH (II:12 and III:28). All family members who participated in the clinical and/or genetic analysis are marked by a star. The numbering of the individuals in the pedigree corresponds to the IDs of the family members in table 1.

The study group consisted of a large German family (S965). A four generation pedigree has been drawn including 83 family members, 59 related to the founder couple (Figure 1).

All 48 living genetically related members were invited to participate in a clinical and genetic evaluation. After informed consent was obtained, 46 members underwent clinical assessment and genetic counseling. EDTA-blood and anticoagulated whole blood were taken for genetic analysis. All relatives were native-born residents of a low altitude area and were assessed in Heidelberg, Germany, at an altitude of ∼100 meters. The study was approved by the Ethics Committee of the Medical Faculty of the University of Heidelberg. All patients gave written informed consent to the study. Informed consent was additionally obtained from the caretaker in case of enrolment of children.

### Clinical procedures

Assessments consisted of recording the family and medical history, physical examination, laboratory parameters including N-type pro brain natriuretic peptide (NT-proBNP), 12-lead ECG, lung function test, arterial blood gases, echocardiography, stress-Dopplerechocardiography (SDE) and cardiopulmonary exercise testing. Manifest HPAH was diagnosed according to the current guidelines [1]. Left heart catheterization and/or CT-scan of the lungs were performed in all patients with suspected left heart or respiratory diseases and when clinically indicated. Right heart catheterization was performed in the living HPAH patients, in the first degree family members and in all family members with suspicion of PH during follow-up.

### Echocardiography

Two-dimensional and Dopplerechocardiographic recordings were obtained using 2.5 MHz Duplex transducers and conventional equipment (Aloka Vario View 2200, Tokyo, Japan) as described previously [19]. Echocardiographic studies were performed by experienced cardiac sonographers (EG, CN), who had no knowledge of the molecular genetic data.

### Stress-Dopplerechocardiography (SDE)

The participants were examined on a supine bicycle ergometer (model 8420; KHL Corp., Kirkland, Washington) as described previously [19]. Systolic pulmonary arterial pressure was estimated from peak tricuspid regurgitation jet velocities according to the equation: PASP = 4 (V)^2^+5 mmHg, where V is the peak velocity (in m/s) of tricuspid valve regurgitant jet, and 5 mmHg is the estimated right atrial pressure. Maximal tricuspid velocity was measured at the highest coherent boundary on the spectral wave form. Signals were considered technically adequate if they had complete envelopes with well-defined borders. In subjects with inadequate Doppler-signals, SDE was repeated within 6–18 months. PASP<25 mmHg at rest and ≤40 mmHg during exercise were classified as normal.^19^ Hypertensive PASP response was declared when maximal systolic pulmonary arterial pressure >40 mmHg was reached during low-dose exercise (up to 125 Watt) in at least one measurement under exclusion of causal hypertensive systemic blood pressure. In patients with hypertensive systemic blood pressure and diastolic dysfunction, treatment for arterial hypertension was initiated.

### Measurements during Hypoxia

Measurements were performed in family members with no known manifest HPAH at baseline = initial visit and in some family members at the next control visit (n = 23). Measurements were performed in a hypoxia-room with a gas mixture of 12% oxygen and 88% nitrogen corresponding to an altitude of 4.500 m. The subjects were examined in supine position and oxygen saturation and heart rate were recorded continuously using a fingertip pulse oxymeter (Ohmeda Biox 3700, Louisville, Colorado, USA). Two-dimensional and Doppler-echocardiographic recordings were performed during baseline in normoxia and at 45, 90, and 120 minutes of hypoxia (F_i_O_2_ = 12%). PASP was estimated as described. For all calculations the mean value of at least 3 TRV measurements was used. Right atrial pressure has been estimated from characteristics of the inferior vena cava [20].

### Right heart catheterization

Right heart catheterization was carried out simultaneously with SDE in all living PAH patients and in 12 first degree family members, using a Swan-Ganz balloon tipped catheter (Baxter, Santa Ana, USA) placed in the pulmonary artery. Pressures at rest and during supine bicycle exercise were recorded using a polygraph (Hellige, Freiburg, Germany). Cardiac output and mixed venous oxygen saturation were obtained at rest and during exercise as described before [19].

### Mutation analysis

Human genomic DNA was prepared from peripheral blood lymphocytes. The complete coding sequence and exon/intron boundaries of the *BMPR2* gene were amplified and analysed by direct sequencing according to Sanger. The complete 5′ untranslated region of the *BMPR2* gene (up to position c.-1270) and the complete intron 5 were also investigated. Primer sequences and PCR conditions are available upon request. Standard DNA sequencing reactions were performed using version 1.1 of Big Dye terminator cycle sequencing kit (Applied Biosystems Inc., Darmstadt) and were analysed on a 3100 Genetic Analyzer (Applied Biosystems Inc., Darmstadt). Screening for larger *BMPR2* rearrangements was performed with the SALSA Multiplex Ligation-dependent Probe Amplification (MLPA) P093-B1 HHT/PPH1 probe mix kit (MRC-Holland BV, Amsterdam, The Netherlands). Mutation nomenclature refers to the NCBI human *BMPR2* nucleotide sequence (Accession number: NM_ 001204) with A of the ATG start codon denoted as +1 and initiator methionine as codon 1.

Furthermore, we analyzed the complete coding sequences of 5 other genes participating in the signalling pathway (*ALK1*, *ENG*, *SMAD1*, *SMAD5*, and *SMAD9*).

### RNA analysis

Lymphocytes from the index patient (III:11) his healthy sister (III:14) were isolated from anticoagulated whole blood and exposed to Epstein - Barr virus (EBV) to induce cell immortalization as previously described [21], [22]. Total RNA was isolated from peripheral blood lymphocytes from all family members and from the cultivated lymphocytes, respectively, by use of a phenol–chloroform method according to Chomczynski and Sacchi [23]. Synthesis of cDNA was performed using Superscript II reverse transcriptase (Life Technologies, Darmstadt) according to manufacturers' recommendation.

Reverse transcription was performed by use of the Transcriptor Reverse Trancriptase Kit according to the manufacturer's recommendations. The following PCR was performed by use of a forward primer located in the untranslated region of exon 1 and a set of reverse primers located in exons 2 to 7 (Roche Diagnostics GmbH, Mannheim; primer sequences and PCR conditions are available upon request). If necessary, RT-PCR products were subcloned by use of the TOPO-TA cloning kit (Life Technologies, Darmstadt). Positive clones were sequenced in both directions.

### Statistical methods

For the analysis of two by two tables with counts lower than 5 Fishers Exact Test was performed using IBM SPSS 20 (SPSS Statistics V20, IBM Corporation, Somers, New York). A p-value <0.05 was counted as significant.

### Genetic counselling and follow-up assessments

In all family members genetic counselling has been performed and clinical follow-up assessment has been recommended in all genetically related family members every 2–6 years. Clinical assessments comprised the same procedures at screening and during follow-up.

## Results

### Clinical assessment

**Figure 2:**
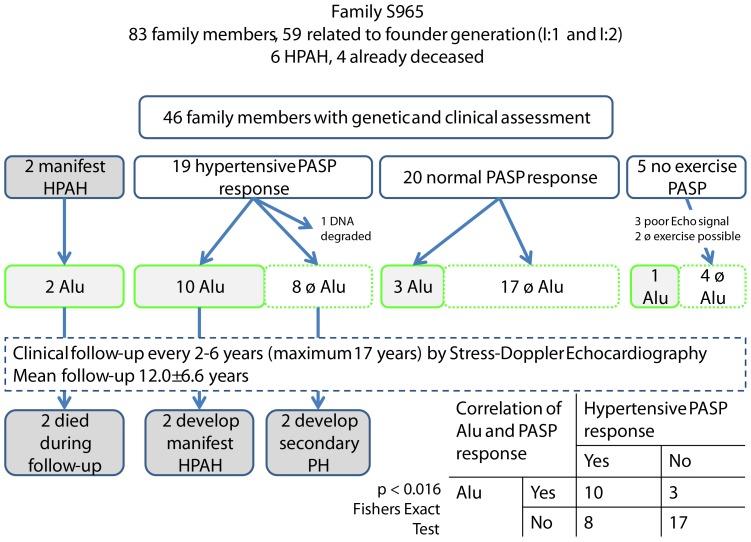
Genotype-phenotype correlations of Alu carriers and PASP during exercise. This figure shows the genotype-phenotype correlation between Alu insertion and PASP during exercise, classified as HR and NR. Only patients who carried both HR and Alu insertion developed manifest PAH during follow-up. Patients with the Alu insertion in the *BMPR2* gene show significantly more frequent Hypertensive response to exercise (10/13 vs. 8/25; Fishers exact test p = 0.016).

The pedigree of the family includes 83 relatives (Figure 1, 2) of which 59 are related to the founder couple (I:1 and I:2). In 1996 when the initial screening started, 11 relatives were already deceased. In total 46 family members participated in the study (21 males, mean age 25.4±13.6 y). Follow-up examinations were performed every 2–6 years with a maximum of 17 years and a mean follow-up time of 12±6.6 years (Figure 2). In 5 family members, PASP during exercise was not measurable. Two patients were not able to perform the cardiopulmonary exercise testing, e.g. family member IV:19, born in the sixth month of pregnancy, presented with spastic tetraplegia and member III:32 could not perform stress echocardiography due to neurological defects. Three family members (III:29, IV:31, IV:32) had poor Dopplerechocardiographic signals. In two family members, elevated PASP response during at least one measurement occurred due to arterial hypertension (III:5, III:9). These patients were classified as normal, as PASP also showed normal increase during hypoxia (III:5, III:9).

### Family members with manifest HPAH

Pulmonary arterial hypertension was already diagnosed in the index patient (III:11) and his female cousin (III:24). Four other family members, who also suffered from HPAH, were already deceased due to right heart failure (II:2, II:10, III:16, IV:16). The index patient died at the age of 57 due to right heart failure, 38 years after diagnosis. His female cousin (III:24) is still alive after initial diagnosis in 1989 (24 years of disease duration until today). Another member (III:28) is still alive after 8 years of PAH. Family members II:2, II:12, II:10, III:16 and IV:16 died within 1 to 5 years (mean: 2.2±1.6 years) after diagnosis, after a short period of disease duration. Family member II:12 died at the age of 69 after the development of lung cancer.

### Family members with normal PASP at rest- hypertensive PASP-response to exercise and/or hypoxia (HR)

In 19 relatives with normal PASP at rest elevated PASP- response to exercise and/or hypoxia was observed (Table 1). Two of the 19 family members developed manifest HPAH during follow-up, four (II:12) and seven (III:28) years after the first screening (Figure 1, 2). Two further family members with HR (III:18 and III:20) developed secondary pulmonary hypertension due to diastolic dysfunction of the left ventricle one year after primary assessments.

**Table 1:** Clinical and genetic assessment in family members.

Pedigree ID	Age at first screening (years)	male/female	BMPR2 (AluYb8 insertion)	Classification due to PASP response during exercise	Echocardiography	Hypoxia	RHC
**III:28**	35	f	y/PAH	HR 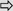 PAH (age 44 y)	✓		✓
II:12	64	m	y/PAH	HR 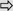 PAH (age 69 y)	✓		✓
III:2	54	f	n.m.	HR	✓	✓	✓
**III:4**	48	f	y	HR	✓	✓	✓
III:7	44	m	n	HR	✓	✓	✓
**III:14**	40	f	y	HR	✓	✓	✓
**III:22**	45	f	y	HR	✓		
**III:31**	18	f	y	HR	✓		
IV:15	26	m	n	NR	✓	✓	
IV:11	10	f	n	HR	✓	✓	
**IV:12**	15	f	y	HR	✓	✓	✓
**IV:13**	13	m	y	HR	✓	✓	
IV:14	10	m	n	HR	✓	✓	
IV:17	17	f	n	HR	✓		
IV:27	16	f	n	HR	✓	✓	
**IV:28**	13	f	y	HR	✓	✓	
**IV:29**	10	f	y	HR	✓	✓	
III:18	43	f	n	HR 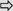 sec. PH	✓		✓
III:20	58	f	n	HR 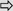 sec. PH	✓		✓
**III:5**	47	m	y	NR	✓	✓	✓
**III:9**	43	m	y	NR	✓	✓	
III:26	38	f	n	NR	✓		
III:33	16	f	n	NR	✓		
IV:1	20	m	n	NR	✓	✓	
IV:2	15	f	n	NR	✓	✓	
IV:3	12	m	n	NR	✓	✓	✓
IV:4	12	m	n	NR	✓	✓	
IV:5	17	m	n	NR	✓	✓	✓
IV:6	14	m	n	NR	✓		
**IV:7**	11	m	y	NR	✓	✓	
IV:9	15	f	n	NR	✓	✓	
IV:10	19	f	n	NR	✓	✓	
IV:8	10	f	n	HR	✓	✓	
IV:18	15	m	n	NR	✓		
IV:21	35	m	n	NR	✓		
IV:22	29	m	n	NR	✓		
IV:23	26	f	n	NR	✓		
IV:30	11	f	n	NR	✓		
IV:33	5	m	n	NR	✓		
III:29	30	m	n	n.m.	✓		
**III:32**	17	m	y	n. m. (seizures)	✓		
IV:19	36	m	n	n. m. (spastic tetraplegia)	✓		
IV:31	9	f	n	n.m.	✓		
IV:32	8	f	n	n.m.	✓		

f =  female, m =  male, n =  no, y =  yes, n.m. =  not measurable, HR =  hypertensive response, NR =  normal response, PAH =  pulmonary arterial hypertension, sec. PH =  secondary pulmonary hypertension, RHC =  right heart catheterization.

One family member (III:4) with HR status recently presented with diastolic dysfunction of the left ventricle. In her last follow-up examination in 2013, she presented with a maximal mean pulmonary arterial pressure of 42 mmHg at 125 Watts and an elevated systemic diastolic pressure of 100 mmHg.

In 11 of 12 patients with hypertensive response (92%), PASP response during hypoxia was equal to PASP response during exercise. One patient who showed normal values during hypoxia was classified as hypertensive responder due to elevated PASP increase during exercise and equal results during exercise right heart catheterization (III:4).

### Family members with normal PASP at rest and during exercise/hypoxia (NR)

Twenty relatives presented with normal PASP response (NR) during exercise. Results of echocardiography were in-line with PASP-increase during hypoxia. No NR-members developed PAH or secondary PH during follow-up.

### Genomic analysis of the *BMPR2* gene

**Figure 3:**
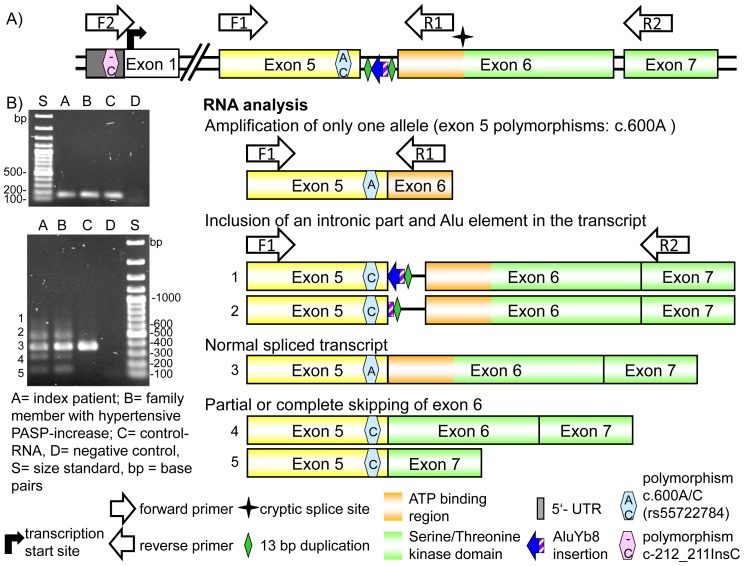
Results of the analysis on RNA level. A) Schematic representation of the genomic region from 5′-untranslated Region (5′-UTR) to exon 7 of *BMPR2*. Indicated are the identified polymorphisms (c.-212_-211insC and c.600A>C) used as markers for the analysis of the *BMPR2* transcript in the index patient, the primer used for the amplification, the location of the inserted Alu element, the duplicated 13 bp region and a potential cryptic splice site (marked by a star with four spikes). B) The upper part of this figure shows the amplified product using the forward primer located in exon 5 with the reverse primer located at the beginning of exon 6. The PCR with primers F1 and R1 resulted in amplification of a single transcript with the polymorphism in exon 5 in apparently homozygous state (c.600A). The other PCR with the same forward primer in exon 5 as above but with another reverse primer located in exon 7 (R2) resulted in amplification of at least five products of different length. Product #3 corresponds to the normally spliced wild type sequence with an apparently homozygous c.600A polymorphism in exon 5; the two larger products (#1 and #2) contained a complete and partial (only left arm) incorporated AluYb8 element, respectively. Furthermore, two smaller products were identified in which the 5′ part of exon 6 (product #4) and the complete exon 6 (product #5) are missing. All aberrant spliced products (#1, 2, 4, and 5) contained apparently homozygous the C-allele of the polymorphism located in exon 5 (c.600C).

After direct sequencing in the index patient no pathogenic mutation in the coding regions for the genes *BMPR2*, *ALK1*, *ENG*, *SMAD1*, *SMAD5*, and *SMAD9* could be identified. In exon 5 of the *BMPR2* gene we found one known polymorphism c.600A>C (p.L200L). This sequence variant was described previously with the frequency of 2.4%.^5^ MLPA analysis did also not reveal any large deletion or duplication in the coding regions of the genes *BMPR2*, *ALK1* and *ENG*. Therefore, we performed screening of the 5′ untranslated region of *BMPR2* exon 1 including the putative promoter region (up to position c.−1270). By this we found only one heterozygous single nucleotide insertion of a cytosine between the nucleotides at position −212 and −211 (c.-212_-211insC). This insertion occurred within a short C-mononucleotide tract consisting of 7 cytosines (Figure 3A). Although we could not confirm the presence of this variant in a cohort of unrelated German controls (n = 86) its polymorphic nature cannot be excluded. PCR experiments performed in relatives of the index patient indicated that both derived sequence variants, the C insertion in 5′ UTR and the A>C polymorphism in exon 5, are located on the same allele. We identified these two variants also in further unrelated patients with I/HPAH (n = 15).

### Analysis of the *BMPR2* transcript

For the investigation of the *BMPR2* transcript in the index patient we used these two heterozygous sequence variants: c.600A>C and c.-212_-211insC as markers. The analyses were performed with the forward primer (Figure 3A, F2) located in the upstream 5′-untranslated region of exon 1 in combination with reverse primers located in exons 2 to 5. This resulted in PCR products containing a C insertion in heterozygous state. In the case of the primer from the distal part of exon 5 the A>C polymorphism was also detected heterozygous (data not shown). This observation indicates transcription of both *BMPR2* alleles. By using a reverse primer located at the beginning of exon 6 (BMPR2-X6-1-RT-R) we could amplify only the wild type allele with respect to both polymorphic variants (Figure 3B, marked as R1 in figure 3b). Further analysis performed with primers located in exons 4 - 7 and 5 - 7, respectively, resulted in amplification of at least five PCR products of different size (Figure 3B). Cloning of the different PCR products and sequencing analysis revealed the presence of the wild type transcript with the entire exon 6, and of four aberrant transcripts. In one, the entire exon 6 was missing (231 bp, position c.622 to c.852, transcript 5) whereas in the second aberrant transcript only the distal fragment of exon 6 (position c.715 to c.852, transcript 4) could be detected. As inferred from the *BMPR2* structure, if translated, the loss of exon 6 does not lead to the change in frame but to the loss of 77 amino acids (amino acids p.208 to p.284). The loss of the first 93 bp of exon 6 in the second abnormal transcript results in deletion of amino acids p.209 to p.238 encompassing the complete ATP binding site of the serine/threonine kinase domain. The two larger products contained both the complete exon 6, 13 bases of the flanking sequence of intron 5 (splice acceptor site), and in addition parts of an Alu element of different length (Figure 3B, transcripts 1 and 2). All aberrant splice products contained the polymorphic c.600C variant whereas in the wild type product the c.600A variant was present indicating that the insertion lies on the same allele as the two polymorphic variants (c.600A>C and c.-212_-211insC). Transcripts 1 and 2 could not been amplified with primer set F1 and R1 presumably due to the AT rich regions close to the primer binding site of R1.

### Screening of the intronic *BMPR2* regions

**Figure 4:**
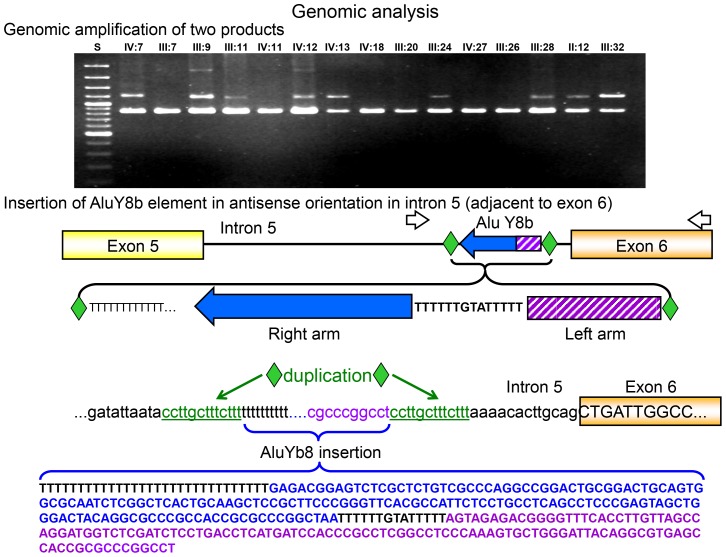
Genomic analysis of the intronic region. The genomic amplification with forward primer located in intron 5 and a reverse primer located in intron 6 resulted in two products of different length identified in several family members (shown is the result of 15 family members). Sequencing analysis of the larger products revealed the insertion of an AluYb8 element with an adjacent duplicated sequence motive in antisense orientation to *BMPR2* in intron 5 (adjacent to exon 6). A schematic representation of the region and the insertion of the Alu element on one allele and the complete sequence of the Alu element, the duplicated sequence and the beginning of exon 6 are shown.

In order to search for the mutation which caused the aberrant splicing of exon 6, we sequenced the adjacent flanking intronic regions. In intron 6 we did not find any mutation. In contrast, with a forward primer located in intron 5 and the reverse primer situated in exon 6 we obtained two PCR products of different length. Sequencing of the longer, gel-extracted PCR product revealed an insertion of an Alu element (AluYb8) in antisense orientation to *BMPR2* 26 bp upstream of exon 6 (c.622-26_-27insAluYb8), which is flanked by a direct duplication of a CCTTGCTTTCTTT sequence (c.622-13_-26; Figure 4). The aberrant *BMPR2* allele is at least 329 bp longer than the normal one. Because of polymerase slippage events during amplification of a long poly-T tract, which is present at the end of this insert, the exact length of the insert could not be estimated.

The Alu insertion was not found in 116 analysed control persons nor reported in the 1000 Genomes Project. Additionally, we did not find the insertion (data not shown).

### Genotype-phenotype correlation

Clinical and molecular genetic information of the family are summarized in Figure 2. In 46 family members clinical assessment and genetic analysis was performed. We found the AluYb8 insertion in 16 family members, four of them with HPAH. According to the inheritance pattern, at least two further deceased family members with manifest PAH were carriers of this insertion (II:2 and II:10, see Figure 1). Additionally, person II:8 carried the insertion but her phenotype could not be determined. For two deceased patients with HPAH neither genetic analysis could be performed nor can the inheritance pattern be used to infer the genetic status. In total 6 of the 15 (13+2 deceased) AluYb8 carriers developed a manifest HPAH resulting in a penetrance of 0.40 (95% CI 0.163–0.677) for this family. If we use the pedigree information and hypothesize that the remaining two HPAH patients (II:2 and II:10) had the AluYb8 insertion as well, the penetrance is 0.47 (8/17, 95% CI 0.230–0.722).

Follow-up data from the echocardiographic measurement of the PASP during exercise was compared between family members carrying the AluYb8 insertion with those family members without the insertion. Ten carriers of the Alu insertion showed a hypertensive PASP response (HR; >40 mmHg) during exercise. A significantly smaller number presented with HR among Alu negative family members (8/25, p<0.016, Fishers exact test, Figure 2). BMPR2 genotype and the PASP status HR/NR were significantly associated. The BMPR2 gene causes PAH and seems also to be associated to hypertensive PASP response.

Both family members that developed manifest HPAH during follow up carried the Alu- insertion and had HR. In contrast, none of the relatives showing normal PASP values during exercise or without carrying the AluYb8 insertion developed manifest PAH during follow-up. Two Alu negative family members developed PH during follow-up due to diastolic dysfunction of the left ventricle which is associated with their left heart disease and systemic arterial hypertension.

## Discussion

This study is the first description of an intronic Alu element insertion resulting in aberrant splicing of the *BMPR2* transcript as the underlying cause for the development of HPAH in a large family, which has been validated by RNA-analysis and was initially overseen by the standard DNA exon sequencing. This result indicates that the proportion of mutations in apparently negative families might be underestimated due to the fact that standard mutation screenings only analyse the exonic regions and short flanking intronic region on DNA level. It highlights the importance of including further methods such as RNA analysis into the molecular genetic work-up of PAH patients. Furthermore, this is the first report an a long-standing follow-up screening of relatives which revealed in this family that it is possible to detect manifest HPAH at an early stage in previously asymptomatic gene carriers. One of the family members who was diagnosed at an early stage responded excellent to PAH-targeted therapy and reminded stable over many years until today. In this family, only mutation-carriers with hypertensive PASP-response to exercise developed a manifest HPAH after several years. Thus, HR is possibly an additional risk factor at least if further underlying causes as high systemic blood pressure and left ventricular diastolic dysfunction have been ruled out.

### Influence of an AluYb8 insertion on the splicing of *BMPR2* transcript

After initial screening of the *BMPR2* coding region [18], [24] by standard screening methods (sequencing and MLPA technique) the family was initially assessed as *BMPR2* mutation negative. However, including the analysis on RNA level by RT-PCR experiments an aberrant splicing of exon 6 was observed which resulted from the insertion of an Alu element from the Y8b subfamily in Intron 5. The AluYb8 insertion in the *BMPR2* gene occurred close to the acceptor splice site, which had markedly increased the distance between this splice site and the branch point in intron 5. In transcript 4 and 5 exon 6 was missing which contains parts of the functionally important serine/threonine kinase domain. We assume, all aberrant splicing products caused either a dysfunctional BMPR2 protein or its complete loss leading to the development of HPAH in this family.

This is supported by reports of intronic Alu insertions causing aberrant splicing previously been published for other diseases [25]–[28]. Furthermore, the inserted Alu element lies in antisense orientation to the *BMPR2* gene. Antisense Alu elements are more likely to cause disease [29]. Lev-Maor et al. [30] previously described in their study a process termed Alu exonization, where retention of antisense Alu elements within the mature mRNA resulted from the introduction of new splice sites from the Alu sequences.

Alu repeats belong to the largest family of mobile elements, the so called short interspersed elements (SINEs), in the human genome. The human-specific subfamilies AluYa, Yb, and Yc belong to the evolutionary youngest SINE elements and are supposed to be still active in terms of *de novo* retrotransposition in modern humans. The current Alu amplification rate is estimated to be in the range of one new insertion per 20–200 human births [31]–[33]. Commonly, the insertion of a new Alu element influences the splicing by changing the open reading frame of the gene. As a consequence, Alu insertions contribute to about 0.1% of human genetic disorders [29], [31], [34]. Alu elements create an approximately 300 bp insertion at any genomic insertion site as seen in our case flanked by a direct duplication of a short genomic region [34]. When an insertion occurs in the middle of an intron or between genes only a minimal effect on genes is observed. In contrast, if an insertion occurs in a coding exon, or near a splice site junction, they are likely to disrupt the proper expression or influence the splicing of a gene [31].

### Genotype-phenotype correlation

The functional analysis of the AluYb8 insertion in the intronic region of the *BMPR2* gene and the clinical follow up show that the AluYb8 insertion is the major cause of HPAH in this family with a penetrance of at least 40–47%. Additionally, the significant association between the presence of the Alu insertion and the PASP status (HR/NR) indicates that the aberrant splicing of the *BMPR2* gene could also be a risk factor for hypertensive PASP response during exercise.

### Early HPAH-diagnosis by regular clinical follow-up assessment

Familial PAH is associated with a worse prognosis. It occurs at an earlier age and progresses more rapidly than non-heritable PAH [35], [36]. Often, healthy family members are interested whether they also have a higher risk to develop HPAH. If a causal mutation is detected in the family, healthy family members can be tested for the familial mutation. They can also be offered intensified medical care that allows early diagnosis and treatment as shown in our family.

Our study shows that HR status is associated to the Alu insertion. We suppose that HPAH and HR may share genetic risk factors.

However, HR should be interpreted with caution as it can also be caused by other diseases e.g. diastolic left ventricular dysfunction in patients with high systemic blood pressure as seen in this family. Thus, for interpretation of an HR, a causal systemic arterial hypertension has to be ruled out.

We observed that both family members that developed manifest HPAH during follow up carried the Alu- insertion and had HR. This is in line with the hypothesis that HR might promote PAH development in mutation carriers, however more evidence from larger samples is needed. These results are also supported by the frequency of HR status in other European PAH-families, showing a significantly higher proportion of HR of 30% compared to 10% in the normal population [19].

However, the clinical course of the family members is still evolving and further cases of PAH and secondary PH may occur in the future. Therefore, further studies and on-going follow-up assessments of PAH families are needed to confirm these results.

## Conclusions

This is the first report of an Alu insertion in an intronic sequence of the *BMPR2* gene leading to aberrant RNA splicing as cause for the development of HPAH in a large German family. Our findings show that analyses on the RNA level increase the rate of *BMPR2* mutation detection, highlight the involvement of aberrant pre-mRNA splicing in the pathogenesis of pulmonary arterial hypertension and extend the mutational spectrum of the *BMPR2* gene. The results support the hypothesis that HR and HPAH share genetic risk factors and additionally that HR promotes PAH development in mutation carriers.
